# Supplementary material for: Data integration across conditions improves turnover number estimates and metabolic predictions
Source: Nat Commun. 2023 Mar 17;14:1485. doi: 10.1038/s41467-023-37151-2 (PMC10023748; doi:10.1038/s41467-023-37151-2)
Supplement: Supplementary file 1 — Supplementary Information [file 41467_2023_37151_MOESM1_ESM.pdf]

Data integration across conditions improves turnover number estimates and metabolic predictions

Wendering and Arend *et al.*

Supplementary Method 1. PRESTO minimization step

By default, PRESTO introduces positive corrections that are added to the k_{cat} values in a pcGEM (see **Methods** section in main text). Here, we describe an optional, second step that can be performed to find negative corrections that also conform with measured exchange fluxes and proteomics data. Therefore, after solving the PRESTO LP, we solve a second optimization problem formulated as follows:

$$\min_{v,\delta,\omega} \frac{1}{|C|}\sum_{j\in C}\omega_j + \frac{\lambda}{|M|-|S_1|}\sum_{i\in M}\delta_i$$

subject to

$$\mathrm{N}\mathrm{v}^{\mathrm{j}} = 0,\ \forall j \in C$$

$$\sum_{i\in \mathrm{GPR}(r)} v_r^j \le \left(k_{cat,i}^{min} + \delta_i\right)\left[E_i^j\right],\ \forall r \in R,\ i \in M,\ \forall j \in C$$

$$\mathrm{v}_{\mathrm{min}}^{\mathrm{j}} \le \mathrm{v}^{\mathrm{j}} \le \mathrm{v}_{\mathrm{max}}^{\mathrm{j}},\ \forall j \in C$$

$$v_{bio}^j \cdot \omega_j \ge \mu_{exp}^j - v_{bio}^j,\ \forall j \in C$$

$$v_{bio}^j \cdot \omega_j \ge v_{bio}^j - \mu_{exp}^j,\ \forall j \in C$$

$$(\varepsilon^{-1} - 1)\cdot k_{cat,i}^{min} \le \delta_i \le 0, \forall i \in M \qquad \textbf{(1)}$$

$$\arg\min\left(\mathrm{k}_{\mathrm{cat}}^{\mathrm{min}}\right) \le k_{cat,i}^{min} - \delta_i, \forall i \in M \qquad \textbf{(2)}$$

$$\delta_k^{k*} - 10^{-9} \le \delta_k \le \delta_k^* + 10^{-9}, k \in S_1 \qquad \textbf{(3)}$$

$$\mathrm{v}_{\mathrm{ex}}^{\mathrm{j*}} - 10^{-6} \le \mathrm{v}_{\mathrm{ex}}^{\mathrm{j}} \le \mathrm{v}_{\mathrm{ex}}^{\mathrm{j*}} + 10^{-6}, \forall j \in C \qquad \textbf{(4)}$$

$$\sum_{j=1}^{|C|}\omega_j \le \sum_{j=1}^{|C|}\omega_j^* + 10^{-9}, \forall j \in C \qquad \textbf{(5)}$$

$$\delta_{\mathrm{inact}} = 0 \qquad \textbf{(6)}$$

$$\omega \le \theta,\ \delta \ge 0.$$

In addition to the PRETO LP for positive corrections of k_{cat} values, the upper bound on δ is changed to zero, while the same fold-change ε is now allowed for the decrease of k_{cat} values (Eq. **1**). The

maximum possible reduction in k_{cat} is further bounded from below by the minimum k_{cat} value in the original pcGEM. Moreover, the positive corrections δ^* from the first step (S_1) are fixed (Eq. **3**). The flux values for exchange reactions are further fixed to the values in the solution to the first LP (v_{ex}^*, Eq. **4**). Eq. **5** bounds the relative error ω from above by the sum of relative errors obtained in the first step ω^*. Finally, the constraint in Eq. **6** prevents corrections to proteins that are only associated to reactions that have zero flux across all conditions in the first solution; it does so by fixing the respective δ to zero. The objective is updated by scaling λ by the difference between the number of proteins that were measured across all conditions and the number of non-zero δ in the solution to the first step.

We observed that the sum of relative errors that is returned by the LP cannot be further reduced by introducing negative corrections. The relative errors in the LP are determined while considering protein abundances, which in both the *S. cerevisiae* and *E. coli* pcGEM led to prediction of lower specific growth rates compared to experimental measurements (underprediction). The introduction of negative corrections can, however, only counteract overprediction. Overprediction, i.e. higher predicted specific growth rate than measured, occurred only then excluding protein abundances and using only an upper bound on the total protein content (pool).

Supplementary Method 2. Flux variability analysis

To investigate how the PRESTO corrections influence the solution space of pcGEMs flux variability analysis (FVA)[1] was implemented. Here we solve a set of LPs based on the GECKO implementation [2] according to

$$\forall r \in R \min_{v,e} / \max_{v,e} v_r$$

subject to

$$\begin{gathered}
\mathrm{Nv} = 0 \\
\sum_{e_m \in \mathrm{GPR}(r)} v_r \leq \left(k_{cat_{m,r}} \cdot e_m\right), \forall r \in R \\
\sum_{m \in \bigcup_r GPR(r)} e_m \cdot \mathrm{MW}_m \leq \sigma \cdot f \cdot P_{tot} \\
\mathrm{v}^{\min} \leq \mathrm{v} \leq \mathrm{v}^{\max} \\
v_{bio} \geq 0.9 \cdot v_{bio}^{max} \\
e_i < [E_i], \forall i \in M.
\end{gathered} \qquad (\mathbf{7})$$

GPR stands for gene-protein-reaction rule that associates reactions with underlying genes and proteins. The index *m* iterates over all enzymes in the model irrespective if a proteomics measurement is available. The only change from the original GECKO formulation aside the new objective is the constraint formulated in Eq. **7** where we bound the flux through the biomass reaction to be at least 90% of the maximum obtained from the initial GECKO FBA problem, v_{bio}^{max}.

Supplementary Method 3. Enzyme abundance prediction

To assess the correspondence between enzyme usage prediction according to the different k_{cat} sets generated by GECKO and PRESTO we formulated an LP that minimizes the enzyme pool (amount of used enzyme). By solving

$$\min_{v,e} \sum_{m \in \bigcup_r GPR(r)} e_m \cdot MW_m$$

subject to

$$\mathrm{Nv} = 0$$

$$\sum_{m \in GPR(r)} v_r \leq \left(k_{cat_{m,r}} \cdot e_m\right), \forall r \in R$$

$$v_{bio} = \mu^{exp}, \qquad (\mathbf{8})$$

we obtain the distribution of minimum enzyme abundances, $\mathbf{e}$, required to support growth at the fixes experimentally observed growth rate according to Eq. **8**. We did not use measured uptake rates in this formulation. We note that except for the corrected model after the optional second minimization step of PRESTO, the predicted enzyme abundances assume all enzymes operating at their maximum catalytic capacity. We presented the Spearman correlation between the obtained solution for the distribution of predicted enzyme abundances, $\mathbf{e}$, and the enzyme abundancy measurements in the given condition for two sets of enzymes: (i) over all enzymes quantified in all conditions and used in PRESTO and (ii) over all predicted enzymes assuming non quantified enzymes are not expressed and thus imputing their abundance with zero (Supplementary Figure 16).

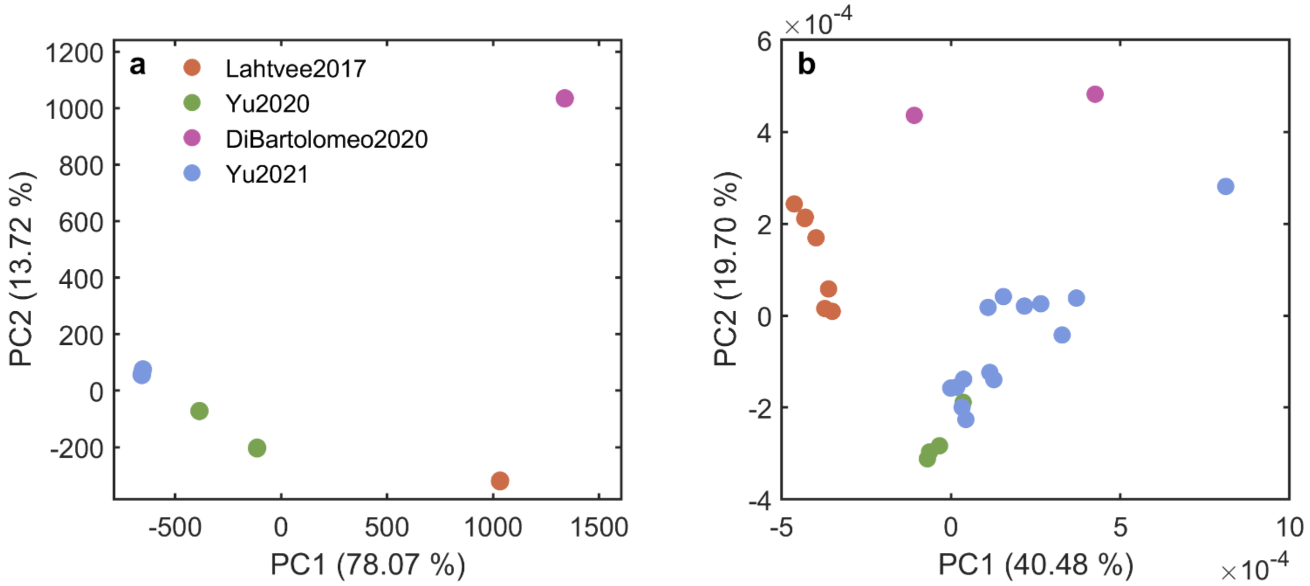

Supplementary Figure 1. PCA results for experimental conditions and protein abundances in the *S. cerevisiae* data set. Principal component analysis was performed using **a** nutrient exchange fluxes and total protein contents and **b** protein abundances from the 27 conditions used in the analysis. The color of the points denotes the study from which the respective samples were obtained, as indicated in the legend.

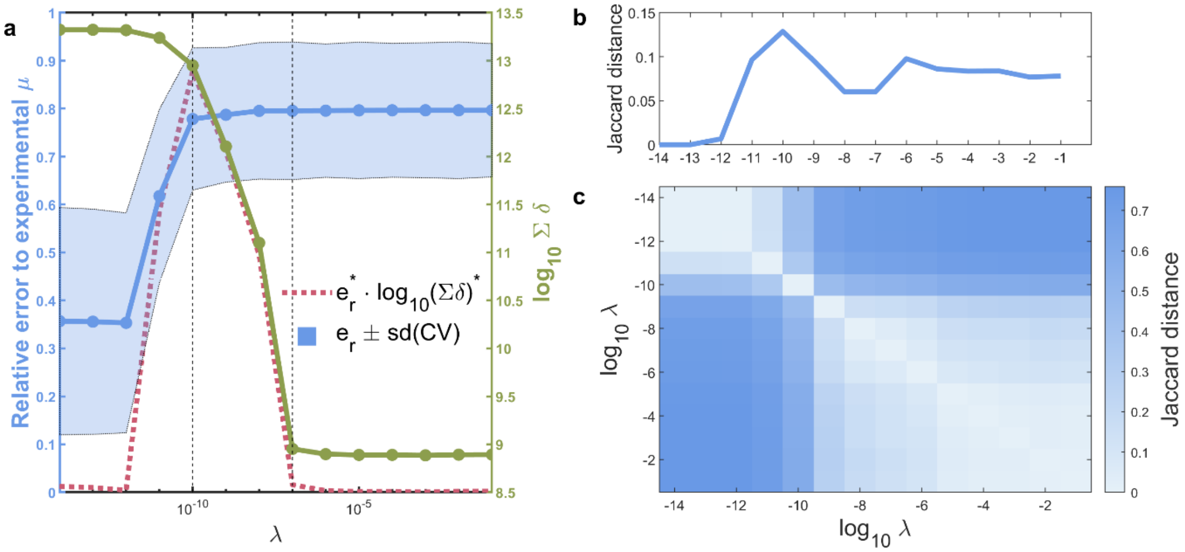

Supplementary Figure 2. Cross-validation to obtain the optimal value for parameter λ using the *S. cerevisiae* pcGEM ecYeastGEM. a Shown are the average relative error (e_r, blue solid line), average sum of added corrections δ (green solid line), and the scoring metric that was used to find the optimal value (red dotted line). Error bands for e_r (light blue) show the standard deviation (sd) across the cross-validation folds (CV). Altogether, 14 values for λ were explored in the range from 10^{-14} to 10^{-1}. The optimal value for λ was determined by the first inflection point of the scoring metric (right dashed vertical line). The left dashed vertical line represents the value at which the sum of δ reaches a plateau. **b** Average Jaccard distance between cross-validation folds over ten iterations for each of the explored λ. **c** Average Jaccard distance between the union of corrected k_{cat} values over ten iterations for each pair of explored λ values.

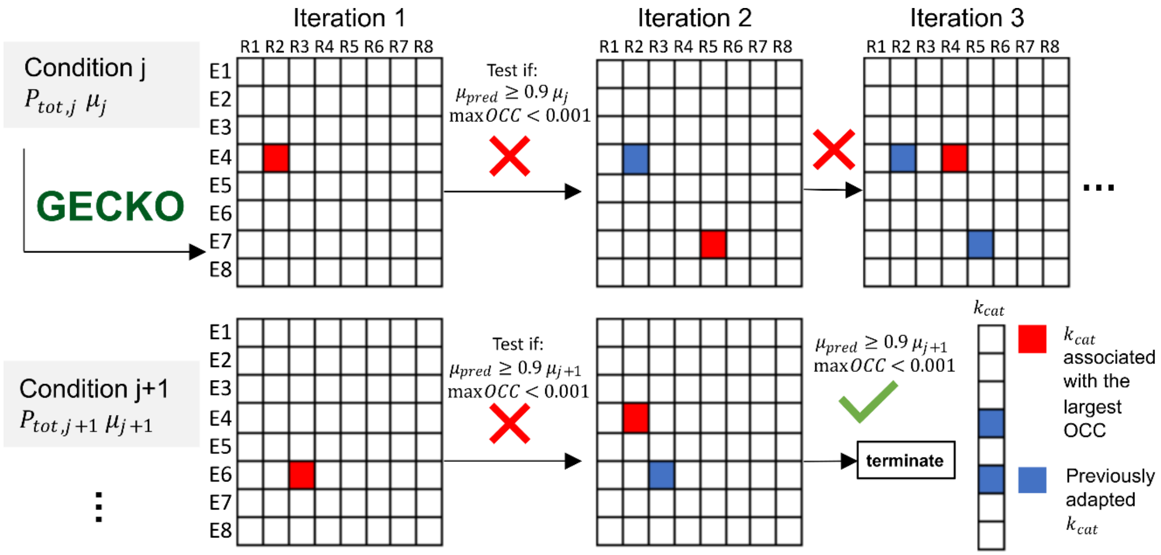

Supplementary Figure 3. Schematic overview of the k_{cat} correction process used by GECKO. The tables correspond to simplified representations of the lower left part of the augmented stochiometric matrix produced by GECKO. To obtain condition-specific models, raw pcGEM models, containing the turnover numbers matched from the BRENDA database, are constrained using the condition-specific total protein content (P_{tot}). If the predicted specific growth rate from FBA (μ_{pred}) is less than 0.9 of the experimentally observed specific growth rate (μ) , each k_{cat}value in the augmented stochiometric matrix is increased by 1000 independently and the effect on the growth rate is measured via the objective control coefficient (OCC). The k_{cat} value associated with the highest OCC is set to the largest value associated with the respective EC number in the BRENDA data base. This procedure is iterated until the predicted growth rate is above the 0.9 threshold or no k_{cat} values are associated with an OCC value greater than 0.001, and results in a set of condition-specific k_{cat} corrections.

a

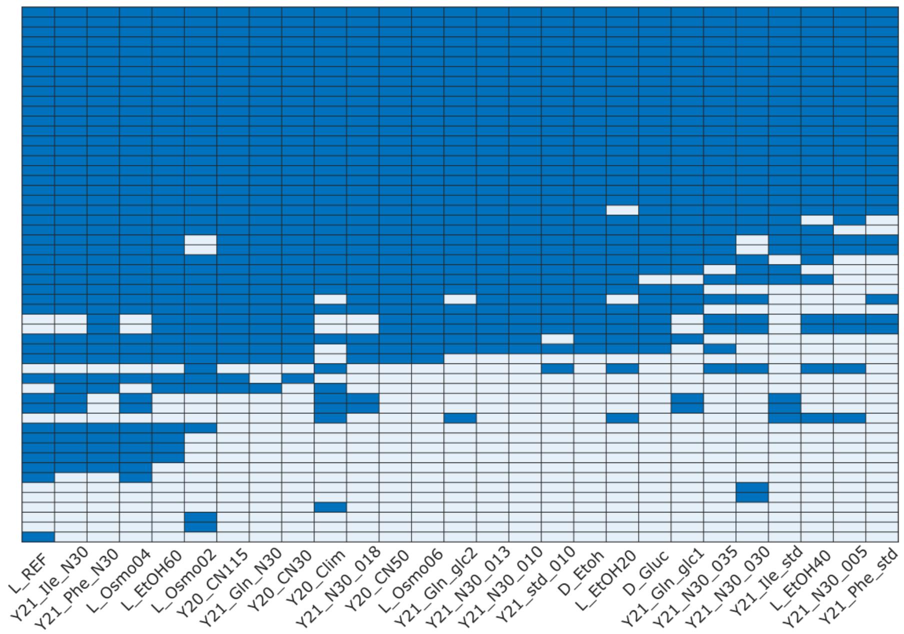

b

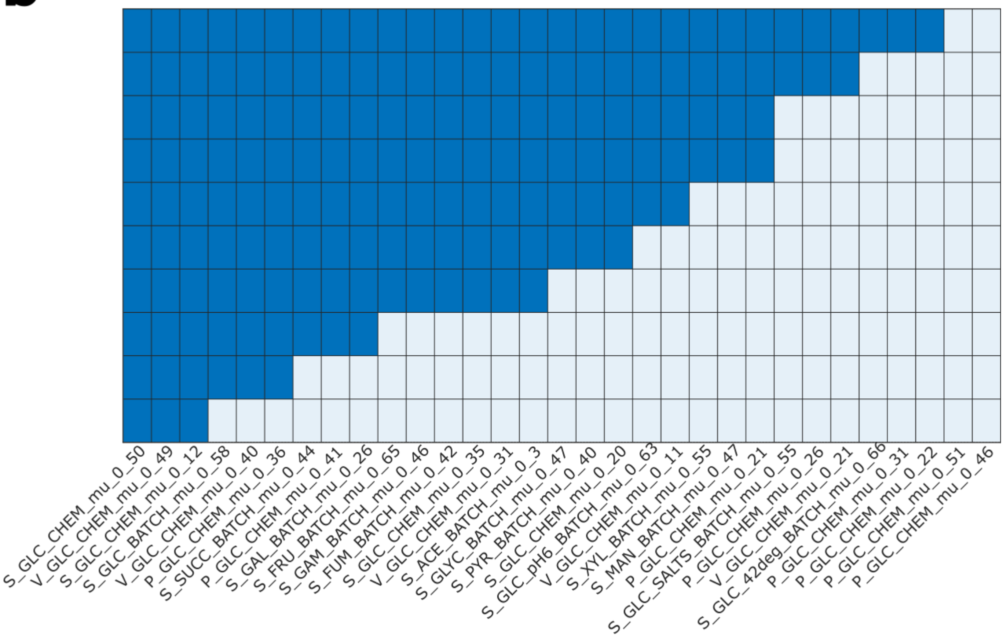

Supplementary Figure 4. Condition-specific GECKO models depict high (*S. cerevisiae*) to perfect (*E. coli*) overlap in ordered corrected k_{cat} sets. The reactions and enzymes whose associated k_{cat} values were corrected by the GECKO heuristic are plotted as heat map. Rows mark the different enzyme reactions and columns indicate the condition-specific GECKO models. Dark blue cells indicate the k_{cat} value was corrected for the respective reaction. Light blue color indicates uncorrected k_{cat} values. The columns and rows were order in descending number of corrected kcats. **a** *S. cerevisiae* models were analyzed. **b** *E. coli* models were analyzed.

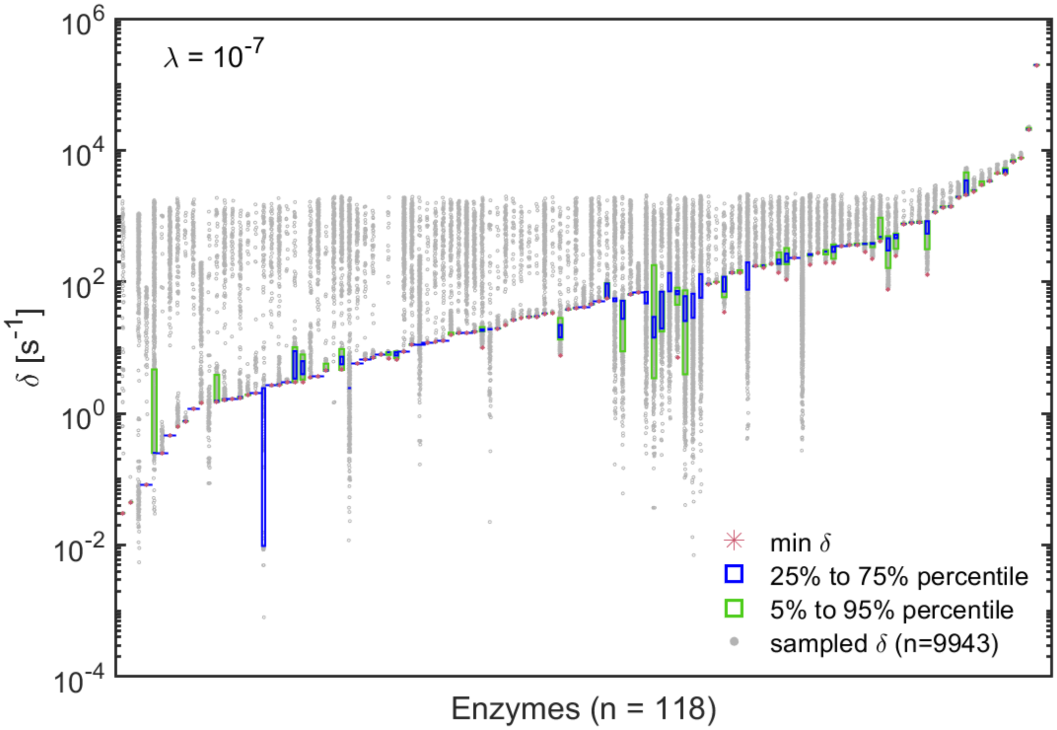

Supplementary Figure 5. Precision of k_{cat} corrections introduced by PRESTO using the optimal λ value for the *S. cerevisiae* pcGEM ecYeastGEM. The minimum and maximum values for each δ were determined by variability analysis in which the relative errors and the sum of corrections, δ, are fixed to values obtained from PRESTO. Using these ranges, 10,000 random points were sampled uniformly across the feasible region (9979 feasible linear programs).

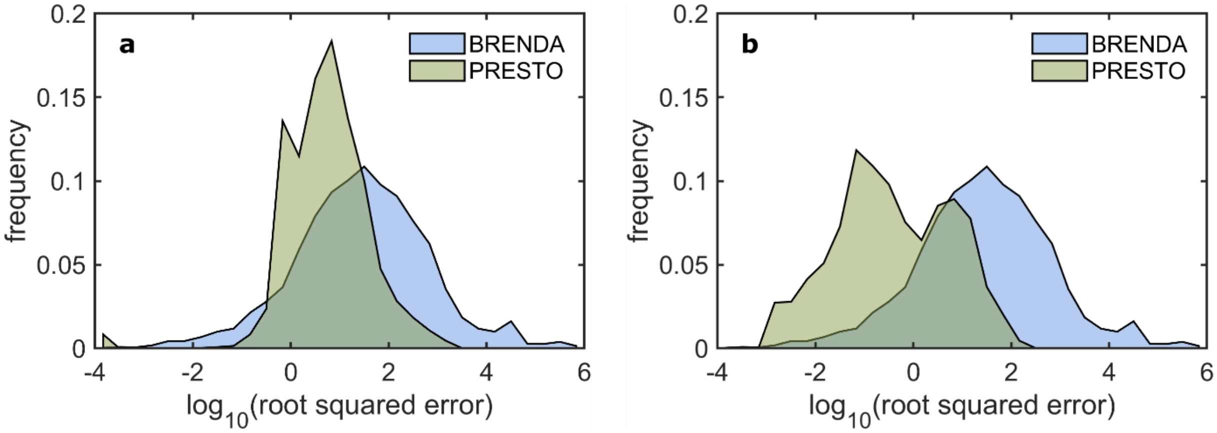

Supplementary Figure 6. Euclidean distance of corrections and $\boldsymbol{k_{cat}}$ values from the respective mean per EC number/protein. The root of squared distances to the average value of sampled corrections from PRESTO (per protein) and k_{cat} values from BRENDA (per EC number). **a** shows the distributions for *S. cerevisiae* ($\lambda = 10^{-7}$) and **b** for *E coli* ($\lambda = 10^{-5}$). To allow for a fair comparison, we only used BRENDA EC numbers for which there are at least three entries for turnover numbers.

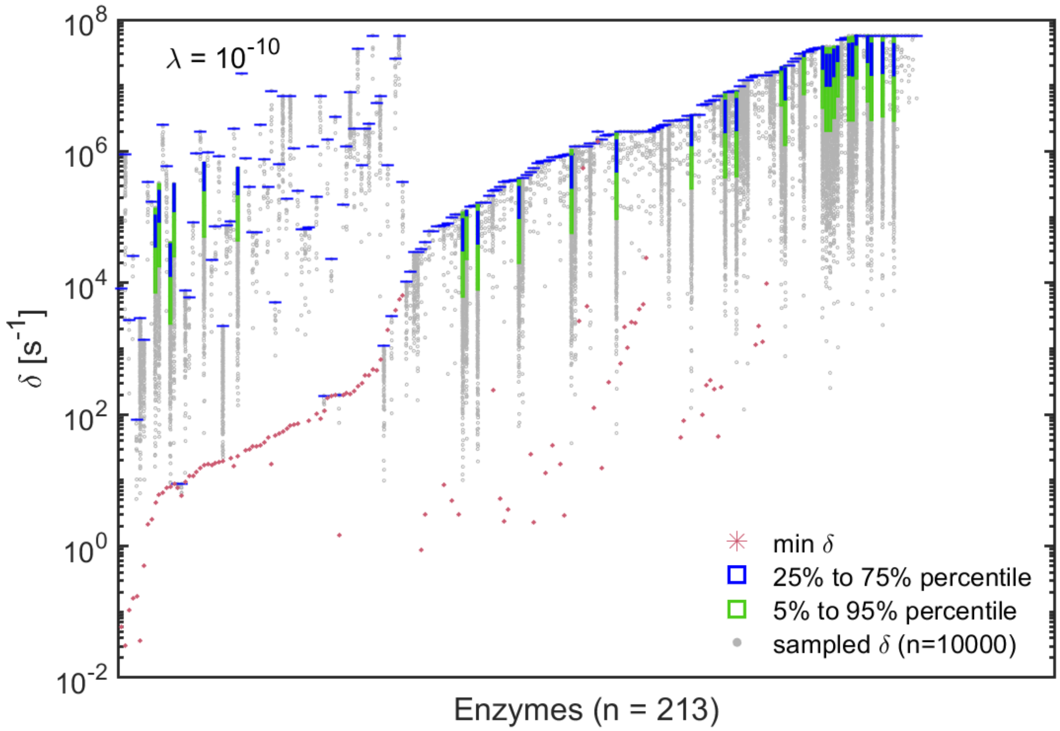

Supplementary Figure 7. Precision of k_{cat} corrections introduced by PRESTO using the λ value for which the average sum of corrections plateaus in the *S. cerevisiae* pcGEM. The minimum and maximum values for each δ were determined by variability analysis in which the relative errors and the sum of corrections, δ, are fixed to values obtained from PRESTO. Using these ranges, 10,000 random points were sampled uniformly across the feasible region. To arrive at feasible optimization programs, the feasibility tolerance for the solver was decreased to 10^{-6}).

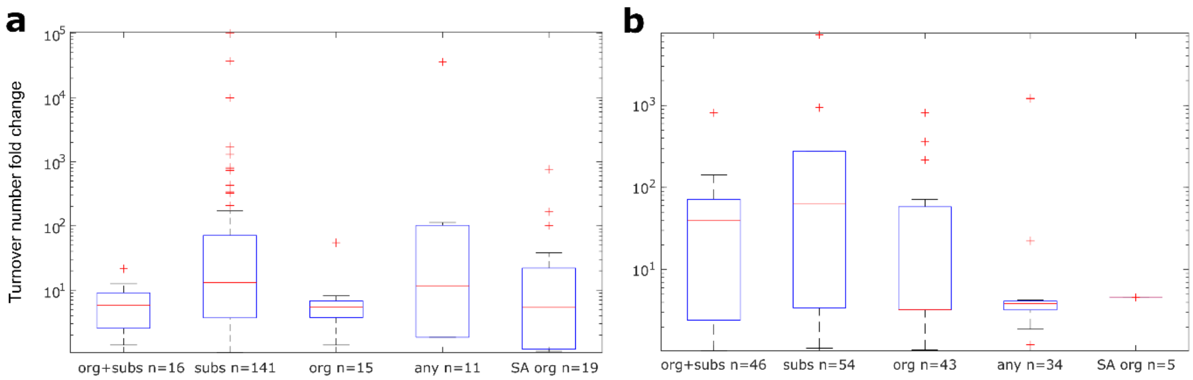

Supplementary Figure 8. Fold-change of PRESTO k_{cat} correction for different GECKO k_{cat} match qualities. For reactions with k_{cat} values corrected by PRESTO in **a** *S. cerevisiae* ($\lambda = 10^{-5}$) or **b** *E. coli* ($\lambda = 10^{-5}$), the quality of the initial BRENDA match in the uncorrected GECKO model was extracted. The boxplots indicate the distribution of turnover number fold-changes in the different matching categories. Categories indicated on the x-axis are exact match of organism and substrate (org+subs), exact match of substrate (subs), exact match of organism (org), only matching EC number (any), exact match of organism in the specific activity database (SA org). Sample sizes for each category per organism are given in the respective x-axis labels. The group of k_{cat} values matched only by EC number in the specific activity database did not have more than four entries in any of the organisms and was therefore omitted in this plot. Middle line and boxes in panels **a** and **b** indicate the median and 25th and 75th percentiles, respectively. Outlier values (plus symbol) are more than 1.5x the interquartile range away from the top or bottom of the box, and whiskers connect the lower or upper quartiles with the non-outlier minimum or maximum.

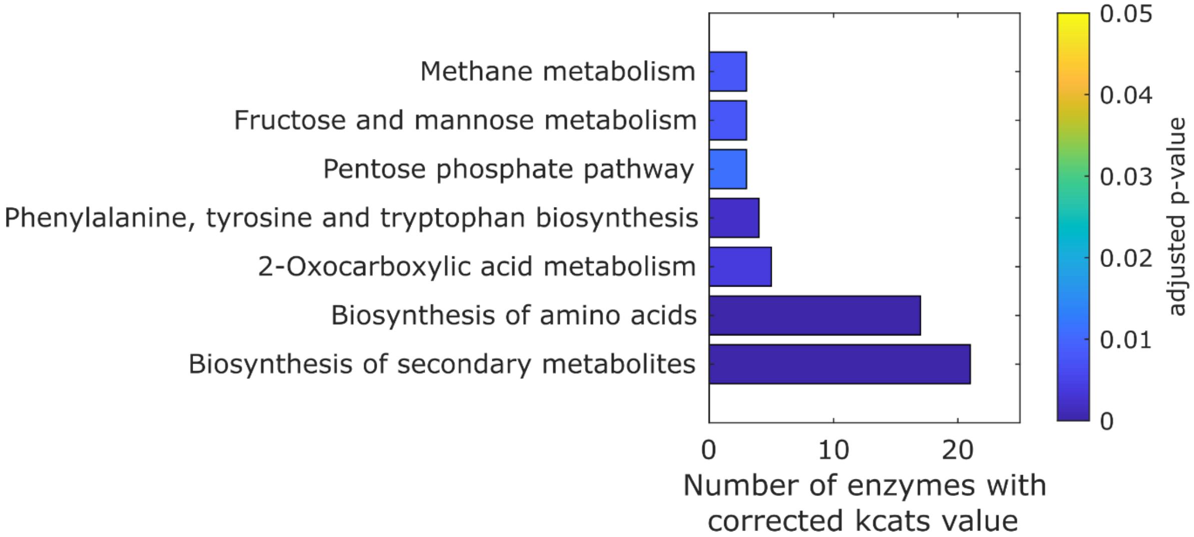

Supplementary Figure 9. KEGG pathway terms significantly enriched in the set of enzymes whose turnover numbers were corrected by GECKO and PRESTO in *S. cerevisiae.* The x-axis gives the number of corrected enzymes linked to the given term. The total number of enzymes in the tested set was 24. Detailed info on the set can be found in Supplementary Data 2. The one-sided p-values were calculated using the hypergeometric density distribution and corrected for multiple hypothesis testing using the Benjamini-Hochberg procedure [3].

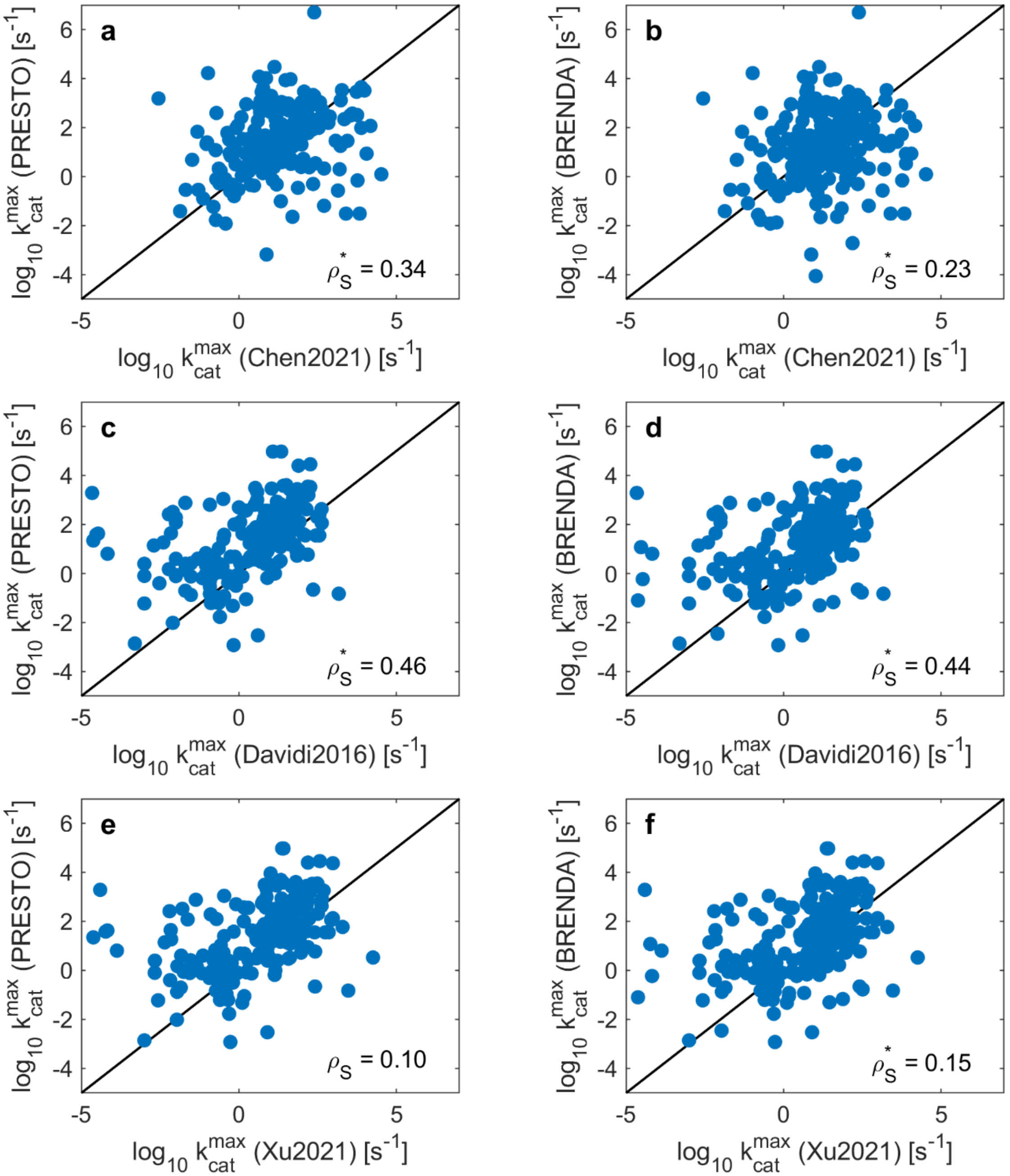

Supplementary Figure 10. Comparison of corrected k_{cat} values from PRESTO with previously published k_{app} values for *S. cerevisiae* and *E. coli*. Comparison of maximum k_{cat} values per reaction with predicted k_{app} values from Chen *et al*.[4] (*S. cerevisiae*; **a,b**),. Davidi *et al*.[5] (*E. coli*; **c,d**), and Xu *et al*.[6] (*E. coli*; **e,f**). The comparison was only performed for reactions associated to homomeric enzymes. The solid line represents hypothetical full correlation. ρ_S: Spearman correlation.

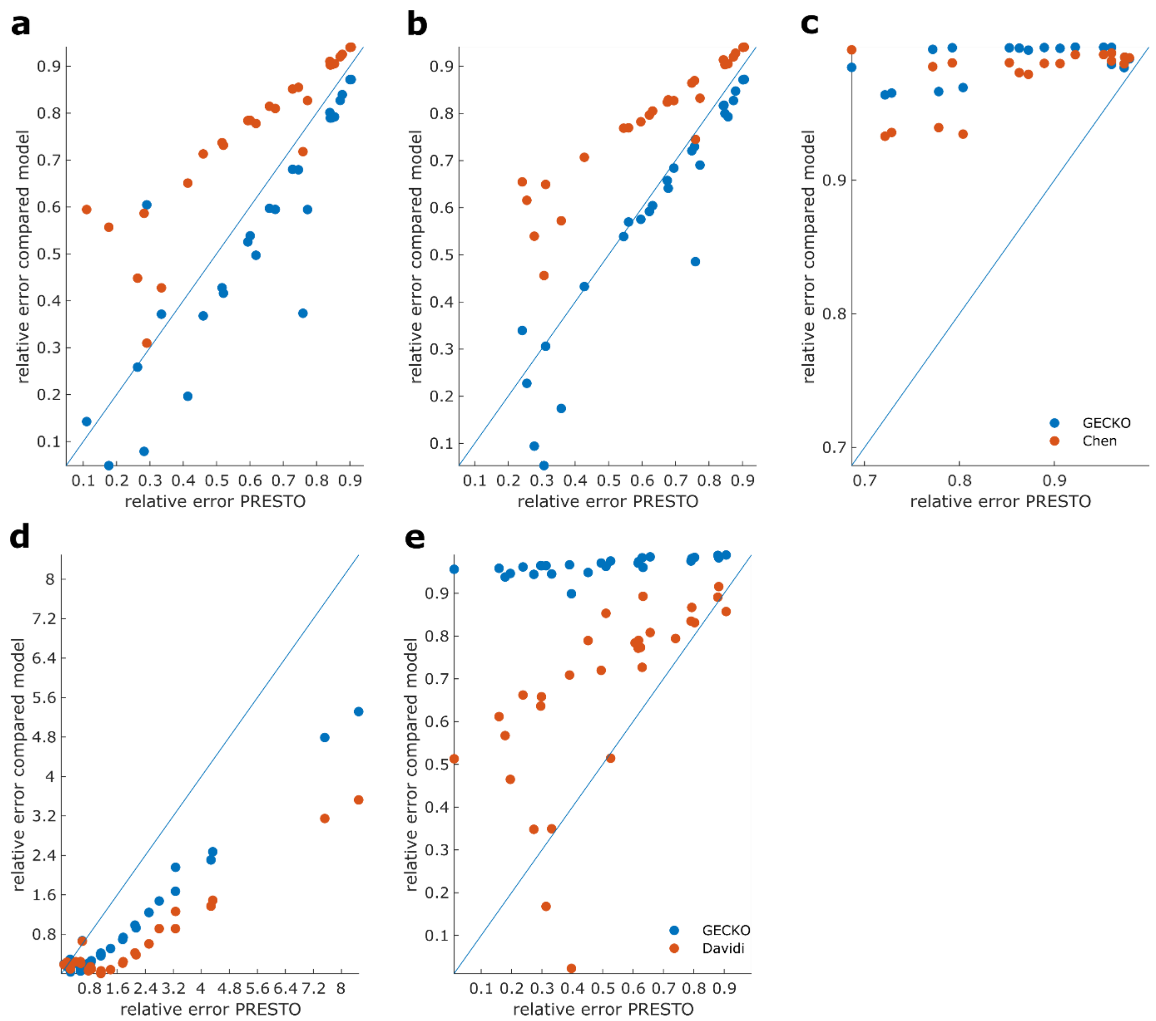

Supplementary Figure 11. Comparison of predicted growth from k_{cat} corrected based on PRESTO, maximum correction from GECKO, and estimated from pFBA. *In vivo* k_{cat} values obtained from pFBA studies of *S. cerevisiae* [4] **a-c** and *E. coli* [5] **d,e** were used to correct initial BRENDA values in the raw pcGEM. To generate a condition-independent GECKO model the maximum k_{cat} over all conditions was used. The y-axis denotes relative error of the GECKO (blue) or pFBA (red) model in comparison to the relative error of the PRESTO model, plotted on the x-axis. **a,d** Only the measured total protein pool was used to constrain the solution and condition-specific uptake rates were bounded by 1000 $\frac{mmol}{h\ gDW}$; **b** in *S. cerevisiae* available measured uptake rates were also considered **c,e** in addition to the previous constraints abundances of enzymes measured in all conditions were used as constraints. The compared pcGEMs in each condition used the same respective biomass coefficients, GAM, $\sigma,$ and P_{tot} values (Methods).

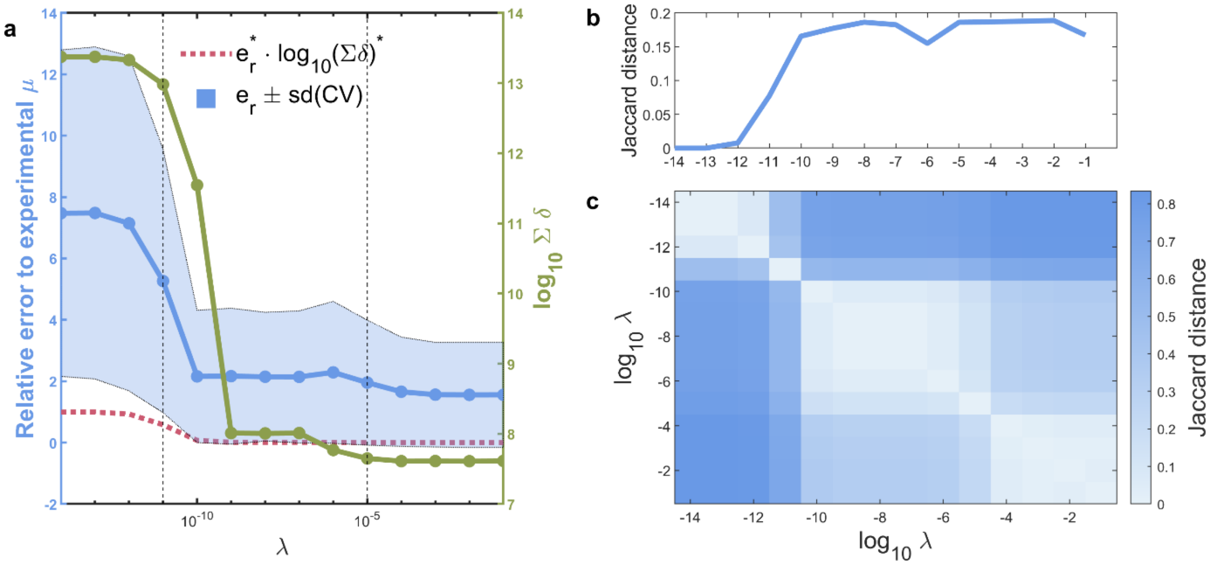

Supplementary Figure 12. Cross-validation to obtain the optimal value for parameter λ for *E. coli* pcGEM. a Shown are the average relative error (e_r, blue solid line), average sum of added corrections δ (green solid line), and the scoring metric that was used to find the optimal value (red dotted line). Error bands for e_r (light blue) show the standard deviation (sd) across the cross-validation folds (CV). Altogether, 14 values for λ were explored in the range from 10^{-14} to 10^{-1}. The optimal value for λ was determined by the first inflection point of the scoring metric (right dashed vertical line). The left dashed vertical line represents the value at which the sum of δ reaches a plateau. **b** Average Jaccard distance between cross-validation folds over ten iterations for each of the explored λ. **c** Average Jaccard distance between the union of corrected k_{cat} values over ten iterations for each pair of explored λ values.

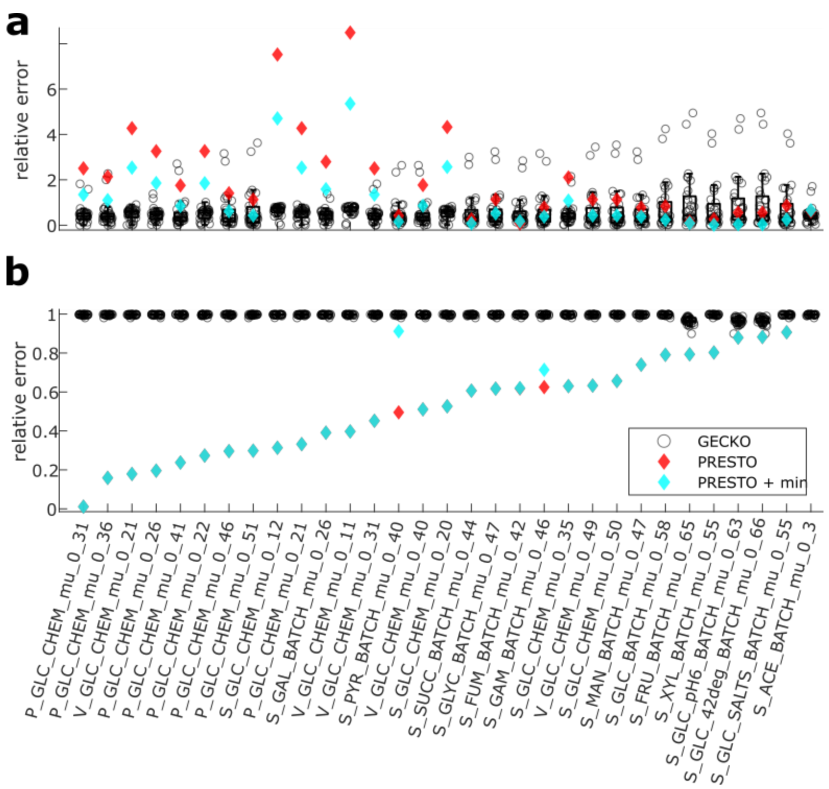

Supplementary Figure 13. *E. coli* model with k_{cat} generated by the two-step PRESTO approach shows lower relative error in growth considering enzyme pool constraint. Condition-specific pcGEMs with corrected k_{cat} values generated by the GECKO heuristic were used to predict the specific growth rate for each condition (n=31, **a** and **b**). The boxplots indicate the distribution of the relative error resulting from each set of condition-specific corrected k_{cat} values obtained from the GECKO heuristic. Relative prediction error from each set is indicated by a circle. The red diamonds show the relative error of predicted specific growth rate from the PRESTO model ($\lambda = 10^{-5}$) by using the single set of corrected k_{cat} values in the respective pcGEM. The cyan diamonds mark the relative error of predicted specific growth rate from the PRESTO models generated using an additional step allowing a reduction of k_{cat} values. **a** Only the measured total protein pool was used to constrain the solution and condition-specific uptake rates were bounded by 1000 $\frac{mmol}{gDWh}$; **b** abundances of enzymes measured in all conditions were used as additional constraints. Missing data points originate from infeasibility of the respective models. The compared pcGEMs in each condition used the same respective biomass coefficients, GAM, σ, and P_{tot} values (Methods). P: Peebo *et al*.[7], V: Valgepea *et al*.[8], S: Schmidt *et al.*[9]. Middle line and boxes in panels **a** and **b** indicate the median and 25th and 75th percentiles, respectively. Outlier values (circles) are more than 1.5x the interquartile range away from the top or bottom of the box, and whiskers connect the lower or upper quartiles with the non-outlier minimum or maximum.

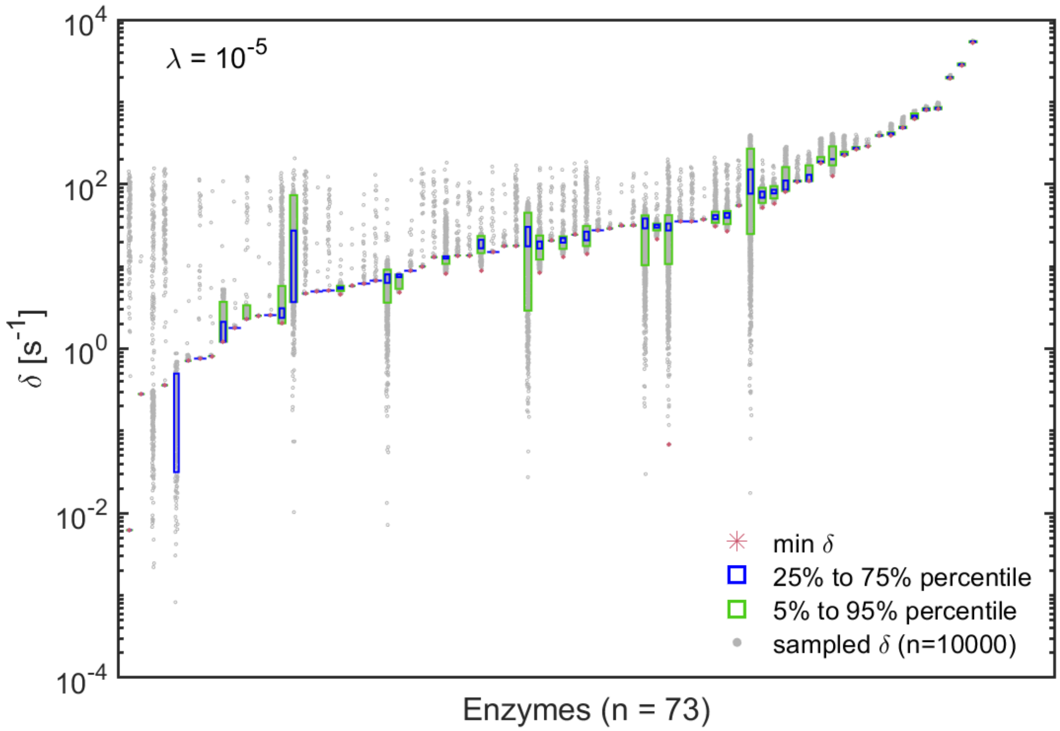

Supplementary Figure 14. Precision of k_{cat} corrections introduced by PRESTO using the optimal λ value for the *E. coli* pcGEM eciML1515. The minimum and maximum values for each δ were determined by variability analysis in which the relative errors and the sum of corrections, δ, are fixed to values obtained from PRESTO. Using these ranges, 10,000 random points were sampled uniformly across the feasible region.

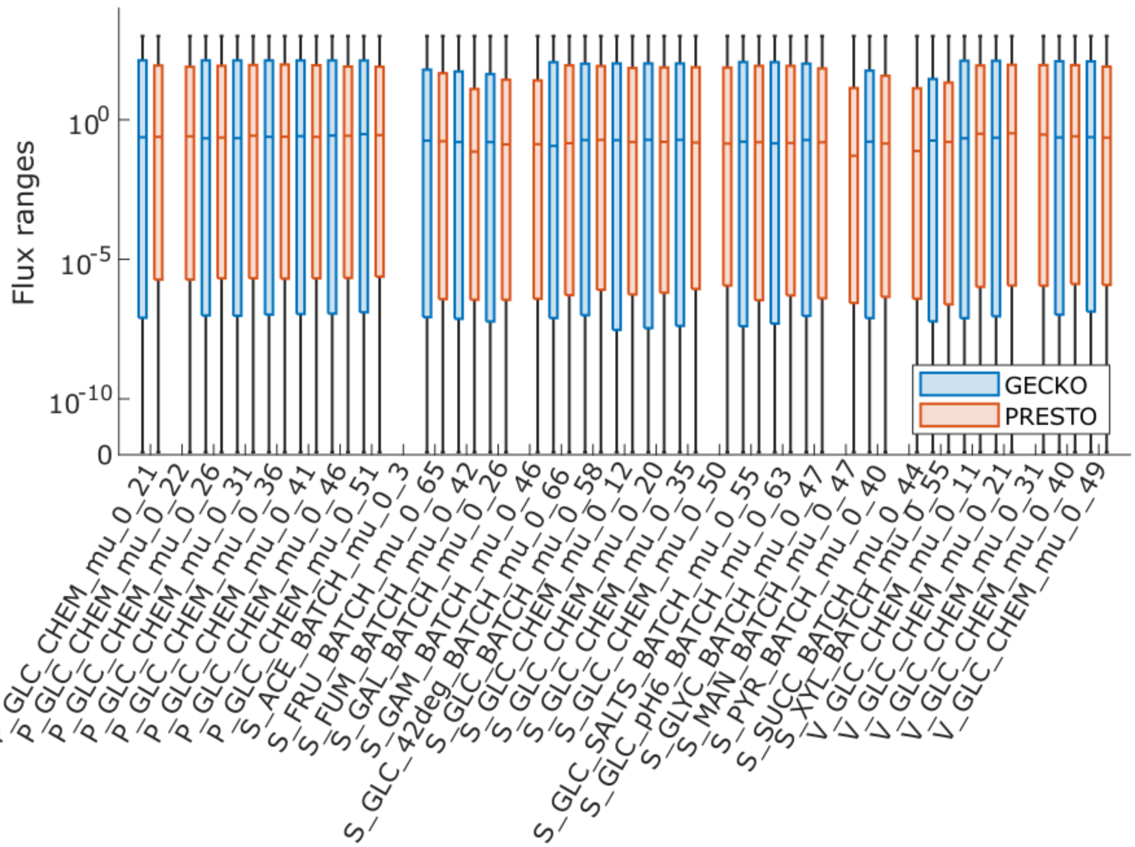

Supplementary Figure 15. Feasible ranges of protein abundance-constrained pcGEMs of *E. coli*. The difference between the maximum and minimum feasible flux through metabolic reactions (n=4830) as determined by FVA in the modelling scenario with experimental measured protein abundance and total enzyme pool constraints are plotted as boxplot on a logarithmic scale (Supplementary Method 2). A condition-independent GECKO model that contained the maximum corrected k_{cat} over all conditions was used. The overall Pearson correlation between ranges in the GECKO and PRESTO models was 0.985. The compared pcGEMs in each condition used the same respective biomass coefficients, GAM, $\sigma,$ and P_{tot} values (Methods). A minimum of 90% of the maximum predicted growth rate was enforced in each condition. P: Peebo *et al*.[7], V: *Valgepea et al.*[8], S: Schmidt *et al*.[9]. Missing bars indicate conditions in which the Gurobi solver was unable to calculate a solution for the FVA problems. Middle line and boxes indicate the median and 25th and 75th percentiles, respectively. Whiskers extend to the minimal and maximal values in the top and bottom end of the boxes.

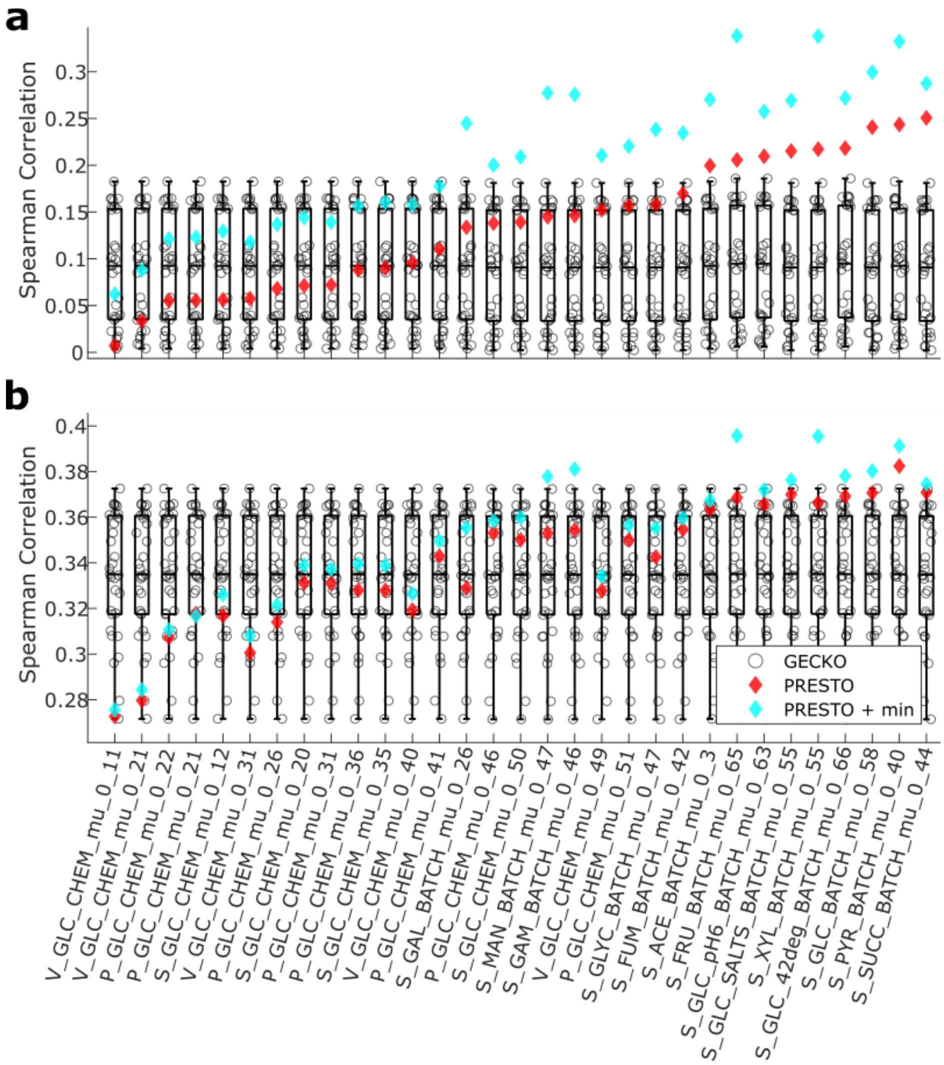

Supplementary Figure 16. Prediction performance of protein abundance based on PRESTO and GECKO pcGEMs of *E. coli*. Condition-specific pcGEMs with corrected k_{cat} values generated by the GECKO heuristic (n=31) were used to predict protein abundances of all enzymes in the eciML1515 model. Condition-specific uptake rates were bound by 1000 $\frac{mmol}{h\ gDW}$ and flux through the biomass reaction was fixed to the experimental growth rate. Further, the total enzyme pool was minimized (Supplementary Method 3). The boxplots indicate the distribution of the Spearman correlation between protein abundances predicted by the GECKO models and the respective proteomics measurements of each condition. Black circles indicate the Spearman correlation of predictions from each set of condition-specific corrected k_{cat} values obtained from the GECKO heuristic. The red diamonds show the Spearman correlation of predictions from the PRESTO model ($\lambda = 10^{-5}$) using the single set of corrected k_{cat} values in the respective pcGEM. The cyan diamonds mark the Spearman correlation of predictions form the PRESTO models generated using the additional step allowing reduction in k_{cat} values. **a** Only proteins that were quantified in all conditions were used to calculate the correlation. **b** All proteins annotated in the pcGEM were used and proteins without quantification in a certain condition were assumed to be absent. The compared pcGEMs in each condition used the same respective biomass coefficients, GAM, $\sigma,$ and P_{tot} values (Methods). P: Peebo *et al.*[7], V: Valgepea *et al.*[8], S: Schmidt *et al.*[9]. Middle line and boxes in panels **a** and **b** indicate the median and 25th and 75th percentiles, respectively. Outlier values (circles) are more than 1.5x the interquartile range away from the top or bottom of the box, and whiskers connect the lower or upper quartiles with the non-outlier minimum or maximum.

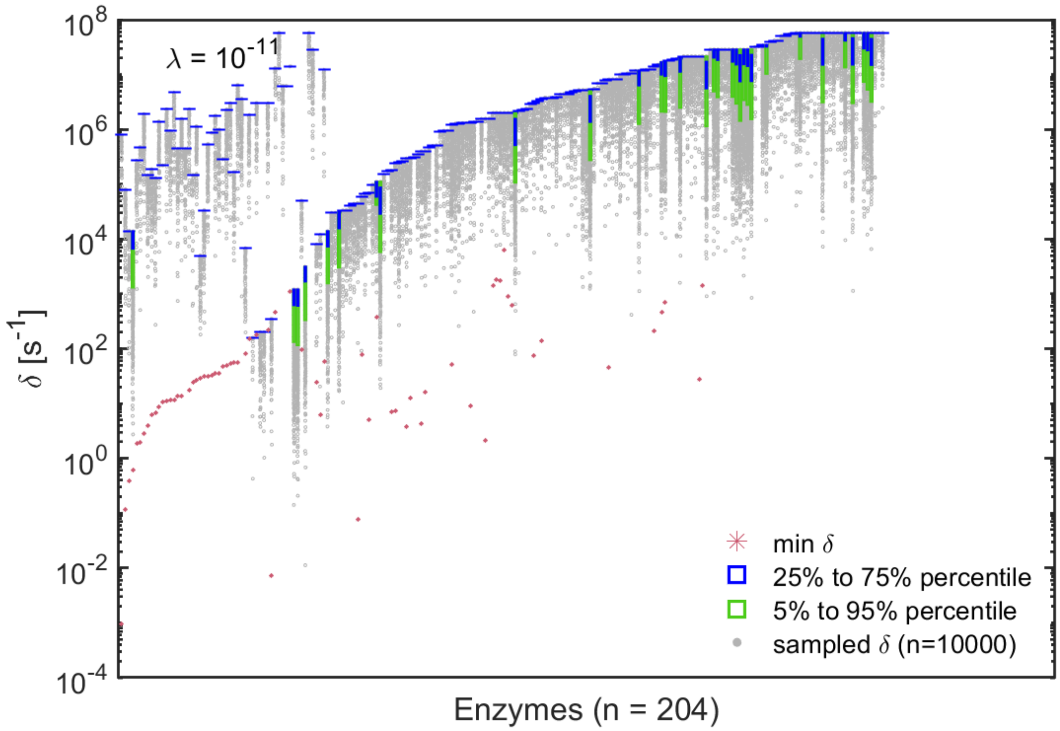

Supplementary Figure 17. Precision of k_{cat} corrections introduced by PRESTO using the λ value for which the average sum of corrections plateaus for the *E. coli* pcGEM. The minimum and maximum values for each δ were determined by variability analysis in which the relative errors and the sum of corrections, δ, are fixed to values obtained from PRESTO. Using these ranges, 10,000 random points were sampled uniformly across the feasible region. To arrive at feasible optimization programs, the feasibility tolerance for the solver was decreased to 10^{-6}).

a

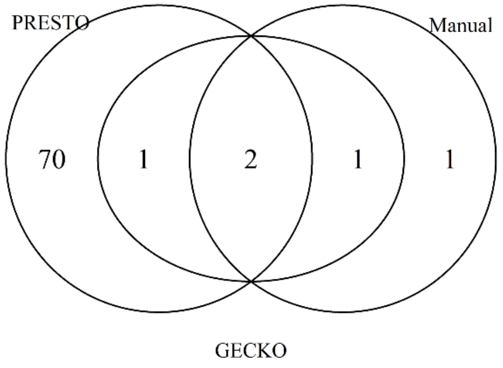

b

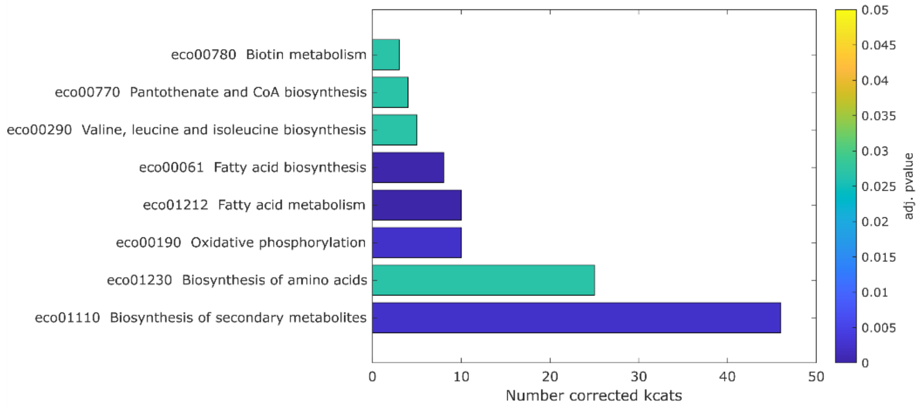

Supplementary Figure 18. Enzymes with k_{cat} values corrected by PRESTO in *E. coli*. a Venn diagram showing the overlap of enzymes whose k_{cat} values were manually corrected [2] ("Manual"), automatically corrected by the GECKO heuristic in any of the conditions ("GECKO") or corrected by PRESTO ("PRESTO", $\lambda = 10^{-5}$) **b** KEGG Pathway terms significantly enriched in the set of enzymes corrected by PRESTO in the *E.coli* model. The x-axis gives the number of enzymes with corrected k_{cat} values linked to the given term. The one-sided p-values were calculated using the hypergeometric density distribution and corrected for multiple hypothesis testing using the Benjamini-Hochberg procedure [3].

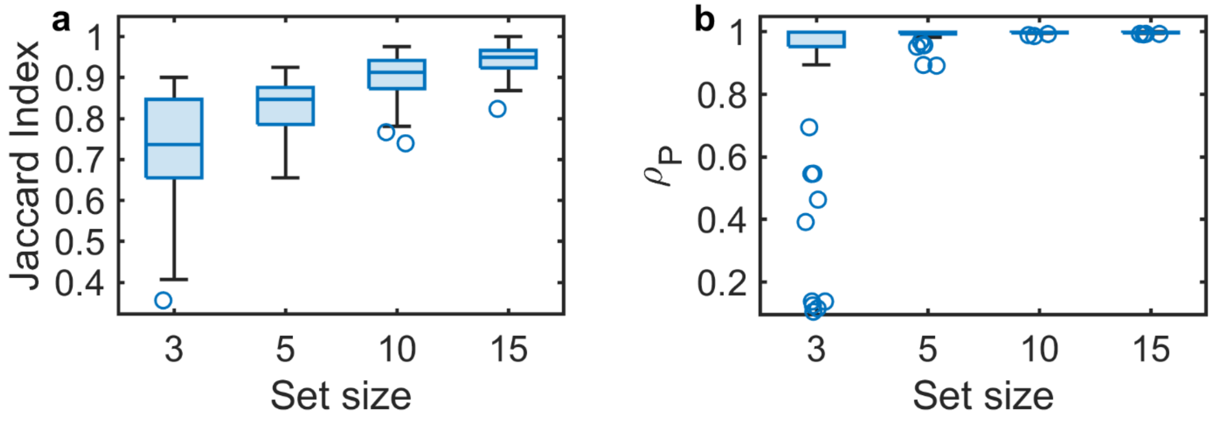

Supplementary Figure 19. Robustness analysis of turnover number corrections for *S. cerevisiae*. The PRESTO approach as used to determine turnover number corrections with different sizes of randomly selected subsets (n=50) of the full dataset comprising 27 conditions. The sets of enzymes with corrected k_{cat} values were compared to the solution obtained with all conditions. This was done by **a** Jaccard Index of the protein identifiers and **b** Pearson correlation of the corrections δ for enzymes whose turnover numbers were corrected using the subset and the full set of conditions. Middle line and boxes indicate the median and 25th and 75th percentiles, respectively. Outlier values (circles) are more than 1.5 x the interquartile range away from the top or bottom of the box, and whiskers connect the lower or upper quartiles with the non-outlier minimum or maximum.

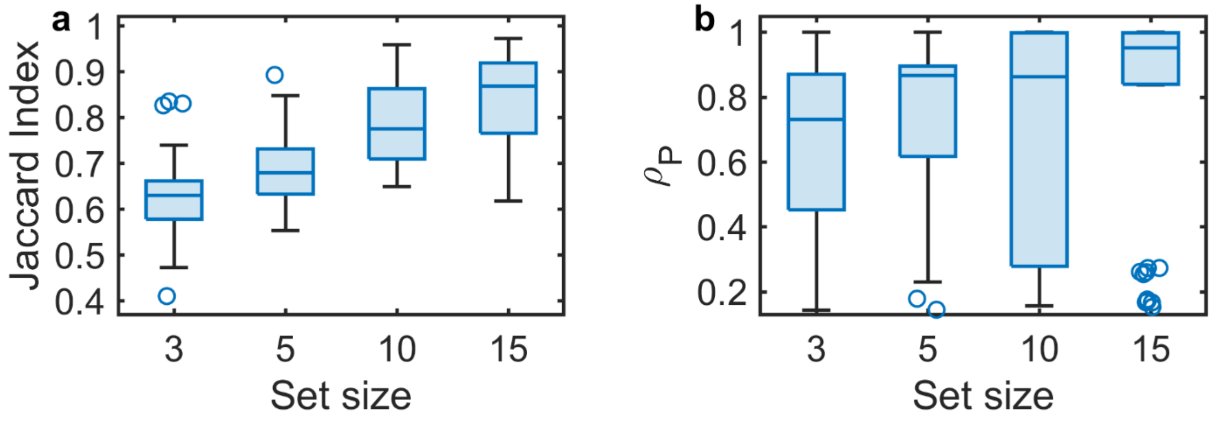

Supplementary Figure 20. Robustness analysis of turnover number corrections for *E. coli*. The PRESTO approach as used to determine turnover number corrections with different sizes of randomly selected subsets (n=50) of the full dataset comprising 27 conditions. The sets of enzymes with corrected k_{cat} values were compared to the solution obtained with all conditions. This was done by **a** Jaccard Index of the protein identifiers and **b** Pearson correlation of the corrections δ for enzymes whose turnover numbers were corrected using the subset and the full set of conditions. Middle line and boxes indicate the median and 25th and 75th percentiles, respectively. Outliers value (circles) are more than 1.5 x the interquartile range away from the top or bottom of the box, and whiskers connect the lower or upper quartiles with the non-outlier minimum or maximum.

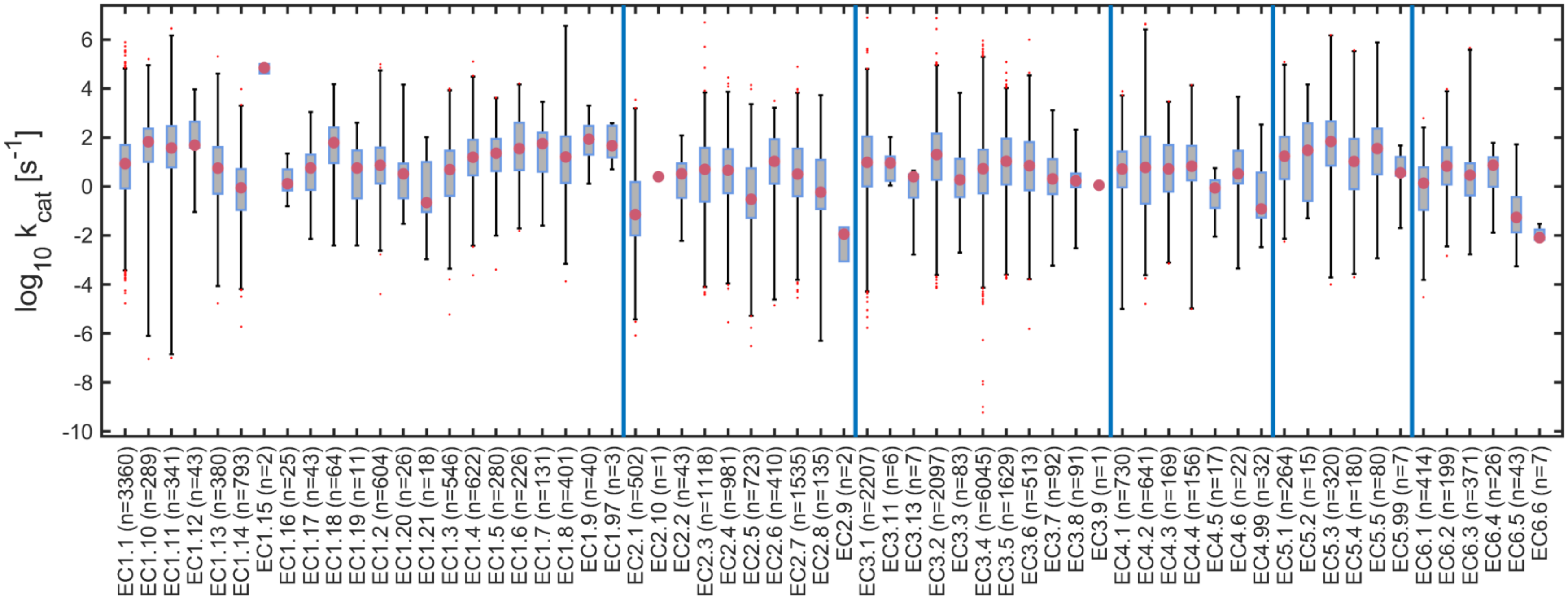

Supplementary Figure 21. Distribution of turnover numbers within EC number classes in BRENDA. The boxplots show log-transformed k_{cat} values for six major EC classes and respective sub-classes as contained in the k_{cat} file provided with the GECKO toolbox [2]. Sample sizes for each category per organism are given in the respective x tick labels. Red dots depict median values, boxes indicate interquartile ranges and whiskers cover 99.3% of data points. Outliers are shown as red stars. Blue vertical lines provide separation between major EC classes.

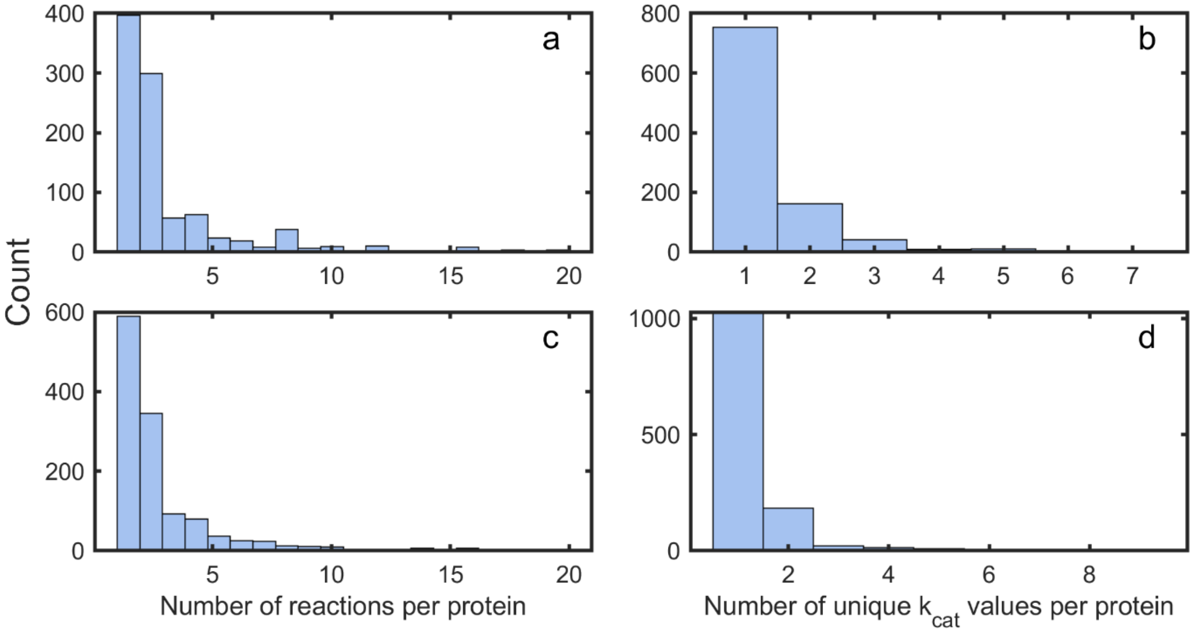

Supplementary Figure 22. Numbers of reactions per enzyme and number of unique $\mathbf{k_{cat}}$ values per enzyme in the *S. cerevisiae* and *E. coli* pcGEMs. (a,c) Histograms of the number of reactions per enzyme (i.e., promiscuity). The histograms were plotted with a range between 1 and 20 reactions per protein. For *S. cerevisiae*, this threshold was exceeded by 31 proteins with a maximum of 384 reactions (*E. coli*: 14 above threshold, maximum: 42) **(b,d)** Histograms showing the number of unique k_{cat} values per enzyme.

Supplementary Table 1. Model performance only using experimental uptake rates and protein abundance but no protein pool as constraints in *S. cerevisiae* pcGEM. Relative prediction error of growth rate by PRESTO model and average relative prediction error and standard deviation of GECKO models. NaN indicates models that were infeasible. sd: standard deviation.

Experimental condition	PRESTO relative error	GECKO average relative error	GECKO sd of relative error
Lahtvee2017_REF	-0.2747	-0.9694	4.09E-09
Lahtvee2017_EtOH20	-0.2171	NaN	NaN
Lahtvee2017_EtOH40	-0.1316	-0.9845	2.32E-09
Lahtvee2017_EtOH60	-0.2432	NaN	NaN
Lahtvee2017_Osmo02	-0.0995	-0.9652	4.48E-09
Lahtvee2017_Osmo04	-0.1040	-0.9639	4.82E-09
Lahtvee2017_Osmo06	-0.1787	-0.9664	2.87E-09
Yu2020_Clim	-0.9820	NaN	NaN
Yu2020_CN30	-0.9736	-0.9843	1.36E-08
Yu2020_CN50	-0.9791	NaN	NaN
Yu2020_CN115	-0.9603	-0.9867	1.12E-08
DiBartolomeo2020_Gluc	-0.9741	-0.9921	5.55E-16
DiBartolomeo2020_Etoh	-0.8220	NaN	NaN
Yu2021_N30_005	-0.6241	NaN	NaN
Yu2021_std_010	-0.8675	NaN	NaN
Yu2021_N30_010	-0.8452	NaN	NaN
Yu2021_N30_013	-0.8910	NaN	NaN
Yu2021_N30_018	-0.9221	-0.9996	3.20E-10
Yu2021_N30_030	-0.9519	-0.9995	3.99E-10
Yu2021_N30_035	-0.9603	-0.9994	5.13E-10
Yu2021_Gln_glc1	-0.7719	-0.9979	2.05E-09
Yu2021_Gln_glc2	-0.7924	-0.9992	7.07E-10
Yu2021_Gln_N30	-0.8468	-0.9993	6.48E-11
Yu2021_Phe_std	-0.8630	-0.9989	1.01E-09
Yu2021_Phe_N30	-0.7999	-0.9987	1.12E-09
Yu2021_Ile_std	-0.8526	-0.9991	7.61E-10
Yu2021_Ile_N30	-0.8659	-0.9974	2.37E-09

Supplementary Table 2. Minimal medium used for simulations with the eciML1515 pcGEM for *E. coli*.

Symbol	Exchange reaction
Na^+	EX_na1_e_REV
P_i	EX_pi_e_REV
Cl^-	EX_cl_e_REV
K^+	EX_k_e_REV
$NH4^+$	EX_nh4_e_REV
Mg^{2+}	EX_mg2_e_REV
SO_4^{2-}	EX_so4_e_REV
MoO_4^{2-}	EX_mobd_e_REV
Mn^{2+}	EX_mn2_e_REV
Ni^{2+}	EX_ni2_e_REV
Zn^{2+}	EX_zn2_e_REV
Cu^{2+}	EX_cu2_e_REV
Ca^{2+}	EX_ca2_e_REV
Fe^{2+}	EX_fe2_e_REV
Fe^{3+}	EX_fe3_e_REV
Cd^{2+}	EX_cd2_e_REV
Co^{2+}	EX_cobalt2_e_REV
H_2O	EX_h2o_e_REV
O_2	EX_o2_e_REV

Supplementary references

1. Mahadevan, R. & Schilling, C. H. The effects of alternate optimal solutions in constraint-based genome-scale metabolic models. *Metab. Eng.* **5**, 264–276 (2003).
2. Sánchez, B. J. *et al.* Improving the phenotype predictions of a yeast genome-scale metabolic model by incorporating enzymatic constraints. *Mol. Syst. Biol.* **13**, 935 (2017).
3. Benjamini, Y. & Hochberg, Y. Controlling the false discovery rate: a practical and powerful approach to multiple testing. *J. R. Stat. Soc. Ser. B* **57**, 289–300 (1995).
4. Chen, Y. & Nielsen, J. In vitro turnover numbers do not reflect *in vivo* activities of yeast enzymes. *Proc. Natl. Acad. Sci. U. S. A.* **118**, 2108391118 (2021).
5. Davidi, D. *et al.* Global characterization of *in vivo* enzyme catalytic rates and their correspondence to *in vitro* kcat measurements. *Proc. Natl. Acad. Sci.* **113**, 3401–3406 (2016).
6. Xu, R., Razaghi-Moghadam, Z. & Nikoloski, Z. Maximization of non-idle enzymes improves the coverage of the estimated maximal in vivo enzyme catalytic rates in *Escherichia coli*. *Bioinformatics* **37**, 3848–3855 (2021).
7. Peebo, K. *et al.* Proteome reallocation in *Escherichia coli* with increasing specific growth rate. *Mol. Biosyst.* **11**, 1184–1193 (2015).
8. Valgepea, K., Adamberg, K., Seiman, A. & Vilu, R. *Escherichia coli* achieves faster growth by increasing catalytic and translation rates of proteins. *Mol. Biosyst.* **9**, 2344–2358 (2013).
9. Schmidt, A. *et al.* The quantitative and condition-dependent *Escherichia coli* proteome. *Nat. Biotechnol.* **34**, 104–110 (2016).
