# Supplementary material for: Data integration across conditions improves turnover number estimates and metabolic predictions
Source: Nat Commun. 2023 Mar 17;14:1485. doi: 10.1038/s41467-023-37151-2 (PMC10023748; doi:10.1038/s41467-023-37151-2)
Supplement: Supplementary file 3 — Description of Additional Supplementary Files [file 41467_2023_37151_MOESM3_ESM.pdf]

Description of Additional Supplementary Files

File Name: Supplementary Data 1
Description: Values of corrected turnover numbers from PRESTO ('PRESTO k_{cat}') and considering the maximum of all k_{cat} corrections from condition-specific GECKO models ('GECKO k_{cat}') in *S. cerevisiae*.

File Name: Supplementary Data 2
Description: Information from UniProt and KEGG servers for the enzymes whose turnover numbers were corrected by GECKO and PRESTO in *S. cerevisiae* (see Fig. 3b and Supplementary Fig. 9).

File Name: Supplementary Data 3
Description: Values of corrected turnover numbers from PRESTO ('PRESTO k_{cat}') and considering the maximum of all k_{cat} corrections from condition-specific GECKO models ('GECKO k_{cat}') in *E. coli*.

File Name: Supplementary Data 4
Description: Information from UniProt und KEGG servers for the enzymes whose turnover numbers were corrected by both GECKO and PRESTO in *E. coli* (see also Supplementary Fig. 18a).
