# Supplementary material for: Data integration across conditions improves turnover number estimates and metabolic predictions
Source: Nat Commun. 2023 Mar 17;14:1485. doi: 10.1038/s41467-023-37151-2 (PMC10023748; doi:10.1038/s41467-023-37151-2)
Supplement: Supplementary file 8 — Reporting Summary [file 41467_2023_37151_MOESM8_ESM.pdf]

nature portfolio

Corresponding author(s): Zoran Nikoloski

Last updated by author(s): Mar 1, 2023

Reporting Summary

Nature Portfolio wishes to improve the reproducibility of the work that we publish. This form provides structure for consistency and transparency in reporting. For further information on Nature Portfolio policies, see our Editorial Policies and the Editorial Policy Checklist.

Statistics

For all statistical analyses, confirm that the following items are present in the figure legend, table legend, main text, or Methods section.

n/a	Confirmed	
☐	☒	The exact sample size (*n*) for each experimental group/condition, given as a discrete number and unit of measurement
☐	☒	A statement on whether measurements were taken from distinct samples or whether the same sample was measured repeatedly
☐	☒	The statistical test(s) used AND whether they are one- or two-sided<br>*Only common tests should be described solely by name; describe more complex techniques in the Methods section.*
☐	☒	A description of all covariates tested
☐	☒	A description of any assumptions or corrections, such as tests of normality and adjustment for multiple comparisons
☐	☒	A full description of the statistical parameters including central tendency (e.g. means) or other basic estimates (e.g. regression coefficient) AND variation (e.g. standard deviation) or associated estimates of uncertainty (e.g. confidence intervals)
☐	☒	For null hypothesis testing, the test statistic (e.g. *F*, *t*, *r*) with confidence intervals, effect sizes, degrees of freedom and *P* value noted<br>*Give P values as exact values whenever suitable.*
☒	☐	For Bayesian analysis, information on the choice of priors and Markov chain Monte Carlo settings
☒	☐	For hierarchical and complex designs, identification of the appropriate level for tests and full reporting of outcomes
☐	☒	Estimates of effect sizes (e.g. Cohen's *d*, Pearson's *r*), indicating how they were calculated

Our web collection on statistics for biologists contains articles on many of the points above.

Software and code

Policy information about availability of computer code

Data collection	ID mapping between gene identifiers to protein identifiers was performed using the Uni UniProt REST API [45] (www.uniprot.org) The KEGG REST API [43] (www.kegg.jp) was used to retrieve pathway information for genes.
Data analysis	All analyses were implemented in MATLAB R2020a/b using functionalities of the COBRA toolbox 3.0 for constraint-based modelling and the GECKO 2.0 toolbox for creating and modifying enzyme-constrained metabolic models. All optimization problems were solved using the Gurobi solver version 9.1.1.<br>All custom computer code generated in this study is available at https://github.com/pwendering/PRESTO.

For manuscripts utilizing custom algorithms or software that are central to the research but not yet described in published literature, software must be made available to editors and reviewers. We strongly encourage code deposition in a community repository (e.g. GitHub). See the Nature Portfolio guidelines for submitting code & software for further information.

Data

Policy information about availability of data

All manuscripts must include a data availability statement. This statement should provide the following information, where applicable:

- Accession codes, unique identifiers, or web links for publicly available datasets
- A description of any restrictions on data availability
- For clinical datasets or third party data, please ensure that the statement adheres to our policy

The protein abundance data used in this study haven been previously published and compiled in Davidi et al. [12], Xu et al. [14], Chen & Nielsen [15].
The UniProt database [45] (www.uniprot.org) was used for mapping gene ID to protein IDs, and the KEGG [43] (www.kegg.jp) database was used to retrieve pathway information for genes.

Human research participants

Policy information about studies involving human research participants and Sex and Gender in Research.

Reporting on sex and gender	n/a
Population characteristics	n/a
Recruitment	n/a
Ethics oversight	n/a

Note that full information on the approval of the study protocol must also be provided in the manuscript.

Field-specific reporting

Please select the one below that is the best fit for your research. If you are not sure, read the appropriate sections before making your selection.

☒ Life sciences ☐ Behavioural & social sciences ☐ Ecological, evolutionary & environmental sciences

For a reference copy of the document with all sections, see nature.com/documents/nr-reporting-summary-flat.pdf

Life sciences study design

All studies must disclose on these points even when the disclosure is negative.

Sample size	Proteomics data:<br>For kcat correction using the E. coli eciML1515 model, we used protein abundance measurements from all conditions (n=30) that were available in the compilation of data by Davidi et al. (DOI: 10.1073/pnas.1514240113) and Xu et al. (10.1093/bioinformatics/btab575).<br>For the kcat correction using the S. cerevisiae YeastGEM 8.5.0 model, we used all available protein abundance measurements compiled by Chen & Nielsen (10.1073/pnas.2108391118), except for experiments that involved variation in temperature (n = 30 - 3 = 27).<br><br>Sampling of kcat correction factor:<br>A sample size of 10,000 was deemed large enough to show meaningful insights into the distribution of correction factors. Given sample sizes for random sampling that are slightly lower than 10,000 result from infeasible optimization problems.
Data exclusions	Samples from temperature stress conditions in the S. cerevisae were excluded due to the strong confounding effects on enyzme catalytic capacity.
Replication	It was not necessary to replicate the computational simulations (i.e. kcat corrections).<br>If proteomics data from multiple replicates were available for the same condition in the datasets, we used the maximum over all available replicates to arrive at a single protein abundance per experimental condition.
Randomization	Randomization was not relevant in this study. The final kcat correction was performed using all proteomics datasets (except temperature stress conditions), which eliminates the need for randomized allocation into groups.
Blinding	Blinding was not relevant in this study. Data collection from databases and experimental studies is inherently non-blinded. Further, there was no hypothesis to blind to that would have biased the outcome of the study.

Reporting for specific materials, systems and methods

We require information from authors about some types of materials, experimental systems and methods used in many studies. Here, indicate whether each material, system or method listed is relevant to your study. If you are not sure if a list item applies to your research, read the appropriate section before selecting a response.

Materials & experimental systems

n/a	Involved in the study	
☒	☐	Antibodies
☒	☐	Eukaryotic cell lines
☒	☐	Palaeontology and archaeology
☒	☐	Animals and other organisms
☒	☐	Clinical data
☒	☐	Dual use research of concern

Methods

n/a	Involved in the study	
☒	☐	ChIP-seq
☒	☐	Flow cytometry
☒	☐	MRI-based neuroimaging
